# On the Relevance of Volumetric Energy Density in the Investigation of Inconel 718 Laser Powder Bed Fusion

**DOI:** 10.3390/ma13030538

**Published:** 2020-01-23

**Authors:** Fabrizia Caiazzo, Vittorio Alfieri, Giuseppe Casalino

**Affiliations:** 1 Department of Industrial Engineering, University of Salerno, Via Giovanni Paolo II 132, 84084 Fisciano (SA), Italy; valfieri@unisa.it; 2 Dipartimento di Meccanica, Matematica e Management, Politecnico di Bari, Viale Japigia 182, 70126 Bari (BA), Italy; giuseppe.casalino@poliba.it

**Keywords:** additive manufacturing, surface roughness, laser powder bed fusion

## Abstract

Laser powder bed fusion (LPBF) can fabricate products with tailored mechanical and surface properties. In fact, surface texture, roughness, pore size, the resulting fractional density, and microhardness highly depend on the processing conditions, which are very difficult to deal with. Therefore, this paper aims at investigating the relevance of the volumetric energy density (*VED*) that is a concise index of some governing factors with a potential operational use. This paper proves the fact that the observed experimental variation in the surface roughness, number and size of pores, the fractional density, and Vickers hardness can be explained in terms of *VED* that can help the investigator in dealing with several process parameters at once.

## 1. Introduction

New methods of manufacturing are receiving special interest to pursue specific advantages in comparison with conventional technologies. Specifically, Additive Manufacturing (AM) by means of laser irradiation of powder bed (i.e., Laser Powder Bed Fusion, LPBF) is being evaluated in many industrial fields, including medicine, aerospace, and automotive [1], thanks to the opportunity of producing custom, complex, accurate components with optimized physical and mechanical features [2,3].

As for any other technology in the field of AM, the process is conducted layer by layer: powder is laid over a plate, then the laser beam provides selective irradiation to the cross-section at each slice of the part. Based on the levels of the governing factors, the process can be conducted either in Selective Laser Sintering (SLS) or Melting (SLM) mode [4]. Since many concurrent phenomena are in place and since LPBF is primarily aimed at industries with stringent standards, different methods of in-line monitoring are required and are currently under development [5,6].

At first, one may expect any outcome in LPBF to depend on the leading factors such as laser power, scanning speed, layer thickness and hatch distance [7], the exposure strategies, as well as the build orientations of the parts [8] and the required supports [9]. Other factors, such as the inert gas selection [10], are deemed to have minor impact, although these have been addressed only recently in the literature. As regarding the output of the process, the resulting mechanical properties and the surface quality are of main concern. Indeed, it has been shown that the porosity, in terms of both amount [11] and size [12] of pores, is crucial for any process involving the laser beam [13], since an effect is played on the ultimate tensile strength and the overall hardness [7,8]; interestingly, a proper selection of the levels of the governing factors is the key to prevent possible anisotropy of the mechanical properties [14].

On the other hand, proper surface quality is crucial for shape tolerances, process accuracy and fatigue life [15,16]; indeed, surface roughness is a limiting factor in LPBF even when manufacturing components with internal channels, because the thermal exchange is affected [17]. Although extensive experimental trials are continuously conducted, improper surface features may still limit the application of the component if compared with its machined or casted counterpart [10]; as a consequence, long and demanding post processing methods are required [18,19] and may represent a challenge in case of complex parts [20]. Nevertheless, specific surface patterns are valuable to the purpose of specific applications, e.g., to promote or hinder adhesion and friction. 

Undoubtedly, both the mechanical and the surface properties must be taken into account when performing a proper optimization. At present, although the optimum processing setting for each base metal is often suggested by the machine manufacturer or found via preliminary trials, interest is growing to investigate possible deviations from the target, to improve a specific requirement depending on the final application: in this sense, the optimum solution must be intended as a compromise. For example, although the residual porosity is unwanted in general, a reduced fractional density may be required to some custom-oriented applications as medical metal implants [21], to adjust the stiffness of the final part depending on the loads and promoting regeneration and better apposition of the bone tissue [22]; conversely, low porosity is mandatory to improve both the mechanical properties and the surface quality. In general, this approach is potentially successful, provided that a complete map is available to assess the measure in which endorsing a response results into the detriment of another. Therefore, ongoing works are reported in the literature and are aimed to full understanding the effects of the governing factors in a wide perspective, on mechanical properties, defect, surface texture and microstructure [23,24,25].

In this frame, the paper investigates the surface texture, the porosity, the fractional density and the microhardness as a function of the governing factors, in order to provide a comprehensive study for selecting the proper processing conditions depending on the application. In agreement with the literature, the main factors involved in LPBF have been combined into the Volumetric Energy Density (*VED*), a synthetic index with physical meaning. Several authors have investigated the process in SLM mode using different definitions of *VED* [26,27,28,29] to prove that the fractional density [30] and other responses effectively depend on *VED* which has been already validated [31] as a design parameter for LPBF of stainless steel. Nevertheless, *VED* is not effective to the purpose of a complete description as it fails to model the physics of the melting pool. In this paper, the definition [29] involving laser power *P*, scanning speed *s*, hatch distance *h* (i.e., the overlapping distance between consecutive traces on the same layer) and thickness *t* of the layer has been considered:

(1) $$VED=\frac{P}{h\cdot s\cdot t}$$

Advantages and limits concerning the investigation of the process have been discussed aiming to validate the effectiveness of *VED* as a design parameter. The investigation has been supported by test of normality for each feature and the analysis of variance to assess the statistical significance of *VED*.

## 2. Materials and Methods

In this work, the levels of the processing factors (Table 1) have been selected in a mixed experimental plan, for a given constant layer thickness of 20 µm, in order to investigate the outcome of LPBF as a consequence of different values of *VED*. The thickness of the layer has been taken as a constant, although affecting the *VED* and the response, since it has been set to match the average size of the particles, to the purpose of uniform layering, based on preliminary trials.

**Figure 1:**
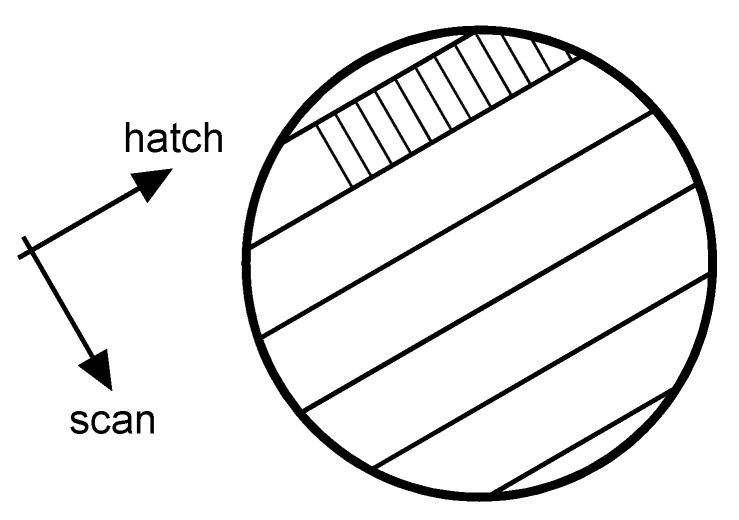
Scheme of the directions of the laser beam in scanning and hatching over a surface.

An EOSINT M270 commercial laser sintering system (EOS, Krailling, Germany), operating in full-melting mode, has been used to manufacture cylinder specimens, 40 mm diameter, 20 mm height; the direction of building is the direction of the longitudinal axis of the cylinder, therefore the need for supports [9] has been prevented. As common practice in LPBF [1], the exposure strategy is based on splitting the cross-section into parallel sectors, 5 mm wide; each sector is scanned by the laser with multiple, overlapping, traces (Figure 1). Moreover, double exposure with the parameters of each processing condition is provided at the contour of the cross-section to the purpose of increasing the accuracy. Layer by layer, the scan direction is rotated by 67° to promote full adhesion of the material and reduce the mechanical anisotropy. It is worth noting that although the manufacturing process is generally based on remelting of the last layer in order to improve the features of the top surface, the common approach has been changed to the purpose of this paper: i.e., the exposed surface has been scanned with the same strategy of any other layer, therefore it is fully representative of the conditions of building. 

Pre-alloyed, virgin, commercial argon gas atomized EOS NickelAlloy IN718 powder of 20 µm mean size, as reported by the manufacturer (EOS, Krailling, Germany), matching to the standard nominal composition of Inconel 718 [32] has been used and laid by a brush recoater, in inert argon atmosphere to prevent oxidation. The temperature of the plate has been set to 80 °C to improve adhesion of the first layer, as well as preventing cracking.

Visual inspections via stereoscopic microscopy have been conducted. To characterize the surface topography, a contact-type roughness tester has been used; specifically, in compliance with ISO standard [33], the sample length and the cut-off wavelength in the Gaussian filter to separate roughness and waviness components of the parts have been set. Moreover, the surface roughness at the top of each sample has been measured along three directions of probe, i.e., the direction of scan (1), the direction of hatch (2) and a 45°-tilted direction (3) with respect to these; three replications have been made for each acquisition. 

Then, the specimens have been cut, mounted, polished and etched with conventional procedures for metallographic preparation of samples [32], to the purpose of further investigation in terms of residual porosity and Vickers microhardness, since a dependence of the mechanical properties as a function of the processing conditions must be assumed.

Porosity has been evaluated in size and amount by an image processing approach: i.e., ten micrographs of 1.20 mm × 0.90 mm size of random sections, orthogonal to the building direction, have been acquired and an algorithm has been implemented to automatically detect the pores as dark spots over a bright surface, based on image segmentation. The number of pores, the corresponding average size, and the residual areal porosity have been computed.

To investigate the effect on the mechanical properties, Vickers testing has been conducted on transverse section, orthogonal to the building direction again; an indenting load of 300 gf has been used for a dwell period of 10 s; specifically, five random cross-sections have been considered, an indentation pattern of 16 tests in a 4 × 4 indentation matrix has been set, a 0.5 mm step has been allowed between adjacent indentations, in compliance with ISO standard [34] for hardness testing on metals. Special care has been taken for the position of indentation in case of significant pore amount, to skip the voids and effectively test the metal.

## 3. Results and Discussion

### 3.1. Surface Roughness and Texture

The mean values of roughness and peak-to-valley height, mean *R_a_* and *R_z_* respectively, have been referred to characterize the surface topography for each processing condition. At first, no densification occurred with the lowest level of *VED* of the plan; the powder was sintered but not fully melted, resulting in the highest roughness. 

**Figure 2:**
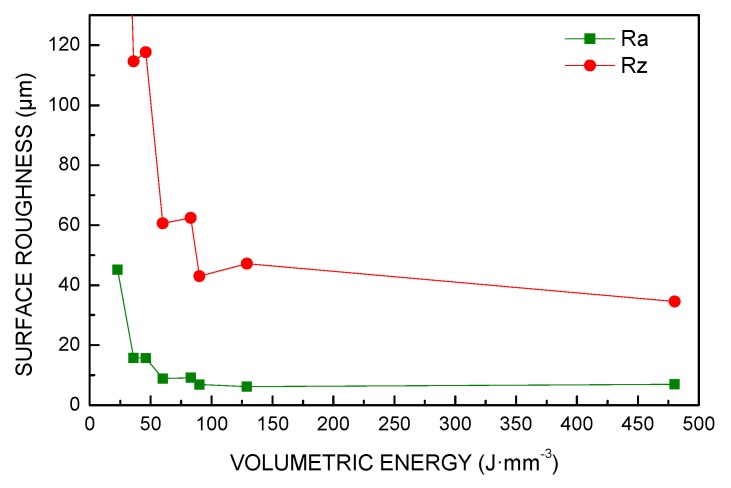
Surface roughness as a function of *VED*.

As regarding the other conditions, effective densification was obtained; interestingly, for each given condition, any dependence of the mean roughness on the direction of probe can be discarded and ascribed to the experimental error; therefore, one may assume that the surface pattern is uniform and the lay resulting from manufacturing is negligible over a surface parallel to the building plate. This is crucial to the purpose of considering a single, average value for the surface roughness (Table 2). Two main findings are displayed. The first one, as-built roughness in LPBF is lower compared to polishing and surface finishing [35] for a given expected dimensional accuracy of 0.01 mm. The second one, any increase of *VED* yields a reduction of roughness (Figure 2).

**Figure 3:**
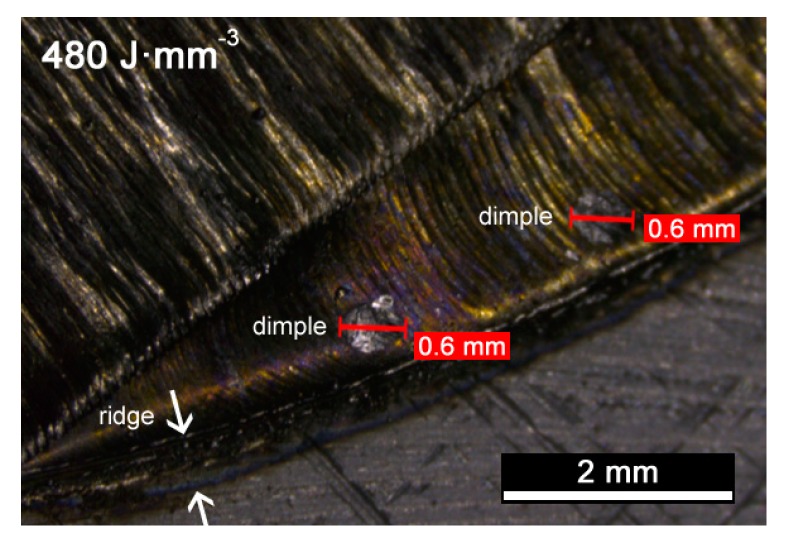
Ridges and dimples at the contour of the cross-section, for *VED* of 480 J·mm^−3^.

**Figure 4:**
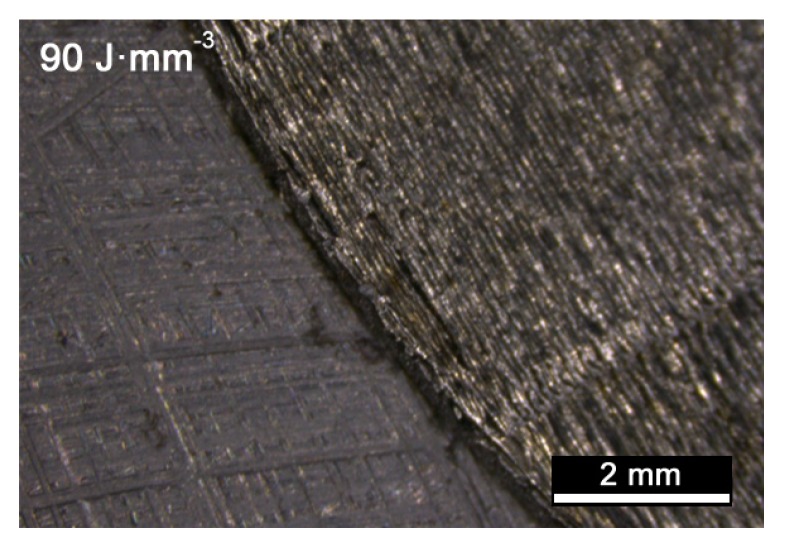
Contour of the cross-section for *VED* of 90 J·mm^−3^.

Nevertheless, exceeding *VED* over a threshold of approximately 90 J·mm^−3^ provided no gain. Indeed, based on the visual inspections on the exposed surface, additional findings must be reported. For example, ridges and dimples result at contour when improper processing conditions are set, i.e., as a consequence of high *VED* (Figure 3); these are prevented in a condition of balanced heat input of 90 J·mm^−3^ (Figure 4), instead. 

Contour ridges are thought to be a major source of imperfections as they may lead to failure of the building process due to delamination of the part or improper layering of the powder; moreover, balling may even occur in condition of low energy per unit volume, due to surface tension hindering proper wetting [36], thus producing unwanted inhomogeneities such as agglomerated particles and dimples [5]. During building, these defects may easily degenerate in porosity, as discussed in the following.

### 3.2. Fractional Density

**Figure 5:**
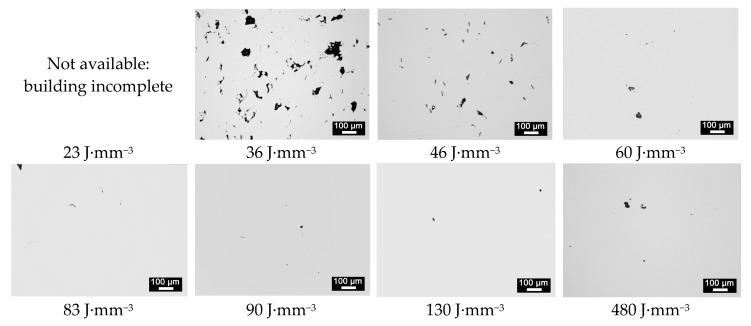
Micrographs of random cross-sections for each condition of *VED*.

*VED* relates to fractional density. Indeed, the total number of pores, their shape and average size are affected (Figure 5). An extreme condition of low heat input resulted in larger, irregular pores where even bits of loose powder locate inside, due to reduced densification rates; conversely, increasing *VED* was favorable to decrease the average number of pores, their average size and the overall void fraction (Table 3). 

**Figure 6:**
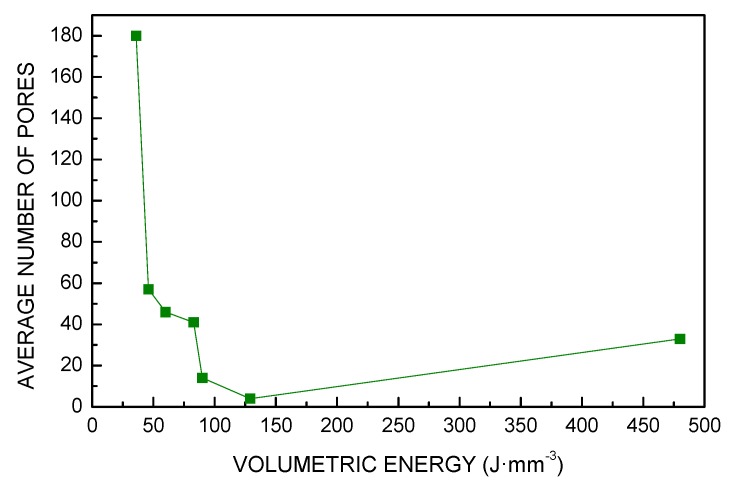
Number of pores as a function of *VED*.

**Figure 7:**
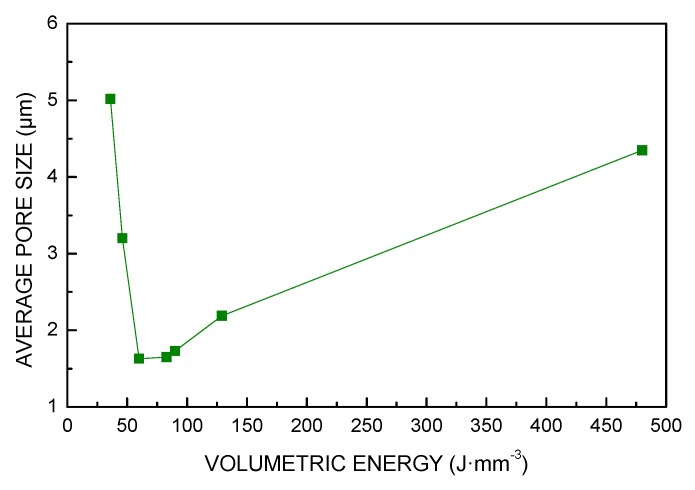
Size of pores as a function of *VED*.

**Figure 8:**
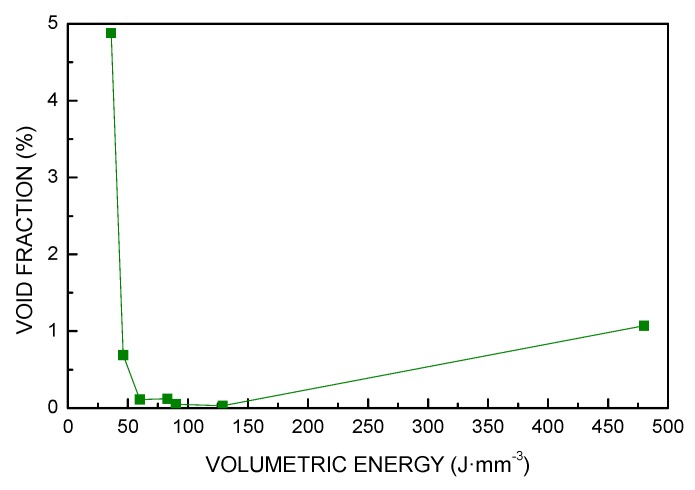
Void fraction as a function of *VED*.

The same trend was found for these responses (Figure 6, Figure 7 and Figure 8): in fact, a 86% reduction of the void fraction, due to both reduced number of pores and decreased pore size, was observed as a consequence of a 67% increase of *VED*; the trend is taken until a critical threshold yielding a slower rate of densification. Indeed, energy input ranging from 60 to 130 J·mm^−3^ resulted in additional improvements in a measure of 72%; nevertheless, further increase of *VED* may be detrimental: interestingly, an increase of the void fraction is found for VED of 480 J·mm^−3^ and must be ascribed to specific defects of layer inhomogeneities, as discussed via visual inspections of the contour of the cross-section.

In agreement with similar findings in the literature [30], an exponential law can be assumed for the fractional density ρ intended as the complement of the residual porosity to 100%:

(2) $$\rho =a-b\exp\left(-c\cdot E_{v}\right)$$

The calibration parameters *a*, *b*, and *c* mainly depend on the particle-size distribution of the powder, although an additional effect could be expected depending on the layering tool. Specifically, *a* denotes the maximum possible theoretical fractional density, whereas *b* and *c* directly depend on the densification rate; all of them must be evaluated on a case-by-case basis. In this case-study, for *VED* in joule per cubic millimeters, the model yields:

(3) a=99.97%

(4) b=15.20%

(5) $$c=0.05\frac{mm^{3}}{J}$$

**Figure 9:**
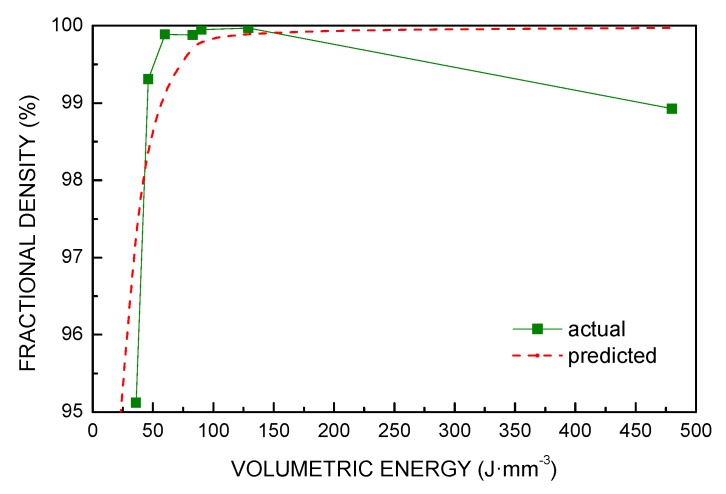
Actual vs predicted fractional density as a function of *VED*.

A 0.7% absolute error is offered by the model in predicting the actual fractional density (Figure 9); the value of the tap density of the powder is approached for null *VED*; the reliability of the model is lost when failures or balling are produced.

### 3.3. Microhardness

**Figure 10:**
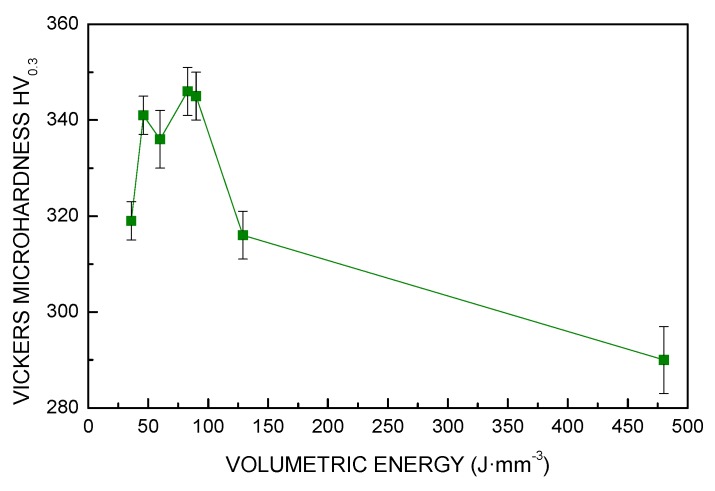
Vickers microhardness as a function of *VED*.

The mechanical properties are expected to be directly dependent on the processing conditions. At first, the formation of pores reduced the overall strength of the part; secondly, different microstructures are formed. Although each given processing condition yields uniform microhardness over random cross-sections, orthogonal to the building plate, a clear dependence on *VED* is in place (Table 4, Figure 10). 

High frequency of pores for low *VED* (i.e., 36 J·mm^−3^), prevented proper testing of the base metal; indeed, the average size of the indentation is larger than the average distance between adjacent pores, therefore lower hardness resulted here. As regarding the other conditions, each indentation is made on full metal, therefore one may assume the values are not affected by the occurrence of porosity, but only by the microstructure which depends on the cooling rate in different processing conditions. Specifically, lower cooling rates due to increased level of energy per unit volume resulted in coarser grains. Otherwise, fine grains were obtained when lower energy was applied. In the range from 50 to 100 J·mm^−3^, considering the extent of the error bars, one may assume that microhardness is not affected by *VED*.

## 4. Statistical Analysis

### 4.1. Assessment of Normality Assumption

Many statistical procedures rely on population normality of the experimental data. Therefore, using a normality test to determine whether to reject this assumption can be an important step in the data analysis. In this paper, the assumption of normality was tested with the Anderson-Darling and the Ryan-Joiner methods. The former compares the empirical cumulative distribution function of the sample data with the expected distribution if the data were normal; the latter assesses the normality by calculating the correlation between the data and the normal scores of the data. If the correlation coefficient is near 1, the population is likely to be normal. The approach is similar to the Shapiro-Wilk normality test [37]. The assumption of normality of data was verified for surface roughness, porosity, fractional density, and microhardness.

The best *p*-value out of the two tests and the range of *VED* for the data used in the test are given (Table 5): the lower the *p*-value, the less the probability that data come from a normal population; i.e., the tests reject the hypothesis of normality when the *p*-value is less than or equal to a cut-off value which has been set to 0.05, as common practice among researchers.

Apart from the size of pores, the hypothesis of normality was verified positively in the range of *VED* between 60 and 480 J·mm^−3^. In this range, the properties of the parts made by Inconel 718 laser powder bed fusion can be predicted using a gaussian distribution of given population parameters (Table 6). As regarding the range between 36 and 60 J·mm^−3^, the process is hindered by imperfect adhesion of the particles, therefore the adaptation to normality is affected.

### 4.2. Analysis of Variance

Based on the results, a dependence on the *VED* is inferred for each of the responses. The analysis of variance (ANOVA) can be used as a definitive exploratory tool to explain the differences in the experimental results. In fact, these can depend either on the experimental error or the explored variable. In the latter case, it is said that the experimental variable has statistical significance, which means that the observed variation is not incidental [37]. The *p*-value of the investigated responses versus *VED* has been evaluated (Table 7); since the *p*-value is a measure of the level to reject the significance of the variable in the explanations of the results, the conclusion is that *VED* is significant for LPBF of Inconel 718, in a range where normality is matched for the responses.

## 5. Conclusions

In this paper, the volumetric energy density relevance to the laser powder bed fusion process was studied. The main findings are listed as follows.

- The increase of the volumetric energy density up to a threshold of approximately 90 J·mm^−3^ results in improved surface features with reduced roughness below 1 µm and fractional density up to 99.97%, with negligible effect on the overall microhardness.
- A critical threshold yielded a slower rate of densification and may degenerate in collapse or delamination of the part, due to ridges and dimples in the cross-section.
- Although it may fail in representing the physics of the process, the volumetric energy density has statistical significance in explaining the variation in the observed experimental results.
- If the VED has statistical significance, it can be used confidently for the process design and optimization, at least in the range of normality for part-quality parameters.

## Figures and Tables

**Table 1:** Processing conditions of LPBF for given layer thickness of 20 µm.

Power (W)	Speed (mm·s^−1^)	Hatch distance (mm)	*VED* (J·mm^−3^)
100	2400	0.09	23
155	2400	0.09	36
100	1200	0.09	46
155	1200	0.11	60
100	1200	0.05	83
195	1200	0.09	90
155	1200	0.05	130
195	400	0.05	480

**Table 2:** Mean values of roughness with corresponding dispersion for each processing condition.

*VED* (J·mm^−3^)	Mean roughness *R_a_* (µm)		Peak-to-valley height *R_z_* (µm)	
	Average	Std. Deviation	Average	Std. Deviation
23	45.12	2.78	285.96	29.89
36	15.82	0.97	114.56	5.75
46	15.77	1.92	117.65	13.57
60	8.92	1.37	60.57	13.80
83	9.08	0.21	62.38	7.04
90	6.84	0.12	43.04	0.42
130	6.22	0.83	47.17	8.10
480	6.99	0.56	34.62	3.79

**Table 3:** Average number of pores, size, and void fraction with corresponding dispersion.

*VED* (J·mm^−3^)	Number of Pores		Size of Pores (µm)		Void Fraction (%)	
	Average	Std. Deviation	Average	Std. Deviation	Average	Std. Deviation
36	180	22	5.02	0.45	4.88	0.57
46	57	21	3.20	0.93	0.69	0.40
60	46	51	1.63	0.95	0.11	0.10
83	41	42	1.65	1.21	0.12	0.07
90	14	2	1.73	0.49	0.05	0.03
130	4	4	2.19	1.80	0.03	0.04
480	33	8	4.35	0.32	1.07	0.35

**Table 4:** Average size of indentation and microhardness with corresponding dispersion.

*VED* (J·mm^−3^)	Size of the Indentation (µm)		Vickers Microhardness HV_0.3_	
	Average	Std. Deviation	Average	Std. Deviation
36	41.8	1.0	319	9.9
46	40.4	0.5	341	8.5
60	40.7	0.5	336	12.3
83	40.1	0.7	346	10.5
90	40.2	0.6	345	10.7
130	41.9	1.0	316	11.5
480	43.9	1.1	290	15.6

**Table 5:** Results of normality test and related range of VED.

Response	*p*-Value	*VED* Range (J·mm^−3^)
Mean roughness *R_a_*	0.53	60–480
Peak-to-valley height *R_z_*	0.05	60–480
Number of pores	0.10	60–480
Size of pores	0.01	60–480
Microhardness	0.36	60–480

**Table 6:** Mean and standard deviation of each response for *VED* in the range 60–480 J·mm^−3^.

Response	Mean	Standard Deviation
Mean roughness *R_a_* (µm)	7.65	1.57
Peak-to-valley height *R_z_* (µm)	49.40	14.79
Number of pores	34.87	25.30
Microhardness (HV)	328.21	3.43

**Table 7:** Results for the ANOVA to test the significance of *VED* in the range 60–480 J·mm^−3^.

Response	*p*-Value
Mean roughness *R_a_*	0.000
Peak-to-valley height *R_z_*	0.028
Number of pores	0.000
Microhardness	0.000
